# Complete Genome Sequence of *Pediococcus pentosaceus* Strain SL4

**DOI:** 10.1128/genomeA.01106-13

**Published:** 2013-12-26

**Authors:** Shruti Harnal Dantoft, Eliza Maria Bielak, Jae-Gu Seo, Myung-Jun Chung, Peter Ruhdal Jensen

**Affiliations:** Systems Biotechnology Group, Department of Systems Biology, Technical University of Denmark, Kongens Lyngby, Denmarka; R&D Center of Cell Biotech Co., Ltd., Gimpo, South Koreab

## Abstract

*Pediococcus pentosaceus* SL4 was isolated from a Korean fermented vegetable product, kimchi. We report here the whole-genome sequence (WGS) of *P. pentosaceus* SL4. The genome consists of a 1.79-Mb circular chromosome (G+C content of 37.3%) and seven distinct plasmids ranging in size from 4 kb to 50 kb.

## GENOME ANNOUNCEMENT

The genus *Pediococcus* is a member of the family *Lactobacillaceae* and is classified as a lactic acid bacterium (LAB), because the main metabolic end product it produces is lactic acid (1). Like other members of the LAB family, *Pediococcus pentosaceus* is a Gram-positive, nonmotile, facultative anaerobe and is acid tolerant. *P. pentosaceus*, along with *P. acidolactici*, is often used as a starter culture in natural and controlled fermentations (2), being a member of indigenous microflora. The strain SL4 was isolated from a traditional Korean fermented vegetable product, kimchi. Based on the 16S RNA sequence, it was taxonomically classified as *P. pentosaceus* and deposited in the Korean Collection for Type Cultures (KCTC) under the accession number KCTC 10297. The strain was shown to inhibit the growth of *Listeria monocytogenes* and *Staphylococcus aureus* via production of a bacteriocin.

The whole genome of *P. pentosaceus* SL4 was sequenced using the Illumina HiSeq 2000 platform (Macrogen, South Korea), yielding 65,886,608 paired-end reads with an average length of 101 bp, amounting to 6,654,547,408 bp and an average genome coverage of ×3,700. A draft assembly was created by assembling the reads *de novo* into 148 contigs, using the CLC Genomics Workbench v. 6.5 (CLC Bio, Aarhus, Denmark). The average contig size was 13,074 kb, with the largest contig being 439,694 kb. The assembled contigs were annotated using the Rapid Annotations using Subsystems Technology (3).

The contigs were analyzed by blast searching at NCBI (4) against the sequence of the closest neighbor of *P. pentosaceus* SL4, *P. pentosaceus* ATCC 25745 (GenBank accession number CP000422). Contigs were further assembled into larger scaffolds, and PCR was used to close the gaps and to obtain a complete circular map of the SL4 chromosome. The assembled map of *P. pentosaceous* SL4 represents a 1,789,138-bp genome, with an average G + C content of 37.3%. This circular genome was annotated by the NCBI Prokaryotic Genome Annotation Pipeline v. 2.2 (released 2013) (http://www.ncbi.nlm.nih.gov/genome/annotation_prok/) (5) upon submission of the genome sequence. A total of 1,817 genes were identified, accounting for 1,709 coding sequences (CDS), 41 pseudogenes, 1 CRISPR array, 15 rRNAs, 51 tRNAs, and 1 noncoding RNA (ncRNA).

The complete genome sequences of SL4 and the type strain ATCC 25745 were aligned using NCBI blast, which showed that 92% of the sequence of SL4 aligned with that of the type strain with 99% sequence identity, and a further analysis of the differences between the strains is warranted. Interestingly, SL4 possesses a large number of plasmids, which poses a challenge in trying to engineer the strain. The plasmid sequences and their roles in the strain are currently under investigation in our lab. A strain of *P. pentosaceus*, IE-3 (accession numbers CAHU01000001 to CAHU01000091), isolated from effluent from the dairy industry (6) has recently been sequenced. The present study combined with the previous genome sequencing efforts could be used to improve our understanding of the effects of diverse ecological niches on genome diversity in this industrially important species.

### Nucleotide sequence accession number.

The complete genome sequence of *P. pentosaceus* SL4 has been deposited in GenBank under accession number CP006854.
